# Supplementary material for: Genetic diversity, mobilisation and spread of the yersiniabactin-encoding mobile element ICEKp in Klebsiella pneumoniae populations
Source: Microb Genom. 2018 Jul 9;4(9):e000196. doi: 10.1099/mgen.0.000196 (PMC6202445; doi:10.1099/mgen.0.000196)
Supplement: Supplementary File 1 [file mgen-4-196-s001.pdf]

SUPPLEMENTARY TEXT

Genetic diversity, mobilisation and spread of the yersiniabactin-encoding mobile element ICE*Kp* in *Klebsiella pneumoniae* populations

Margaret M. C. Lam[1], Ryan R. Wick[1], Kelly L. Wyres[1], Claire L. Gorrie[1], Louise M. Judd[1], Adam W. J. Jenney[2], Sylvain Brisse[3], and Kathryn E. Holt[1#].

Author affiliations:
[1]Department of Biochemistry and Molecular Biology, Bio21 Molecular Science and Biotechnology Institute, The University of Melbourne, Parkville, Victoria, Australia
[2]Department Infectious Diseases and Microbiology Unit, The Alfred Hospital, Melbourne, Victoria, Australia
[3]Institut Pasteur, Biodiversity and Epidemiology of Bacterial Pathogens, Paris, France

#Address Correspondence to Kathryn E. Holt, kholt@unimelb.edu.au
Bio21 Molecular Science and Biotechnology Institute, 30 Flemington Rd, Parkville, Victoria, Australia, 3010.

Index:

ybt* locus diversity in *K. pneumoniae **2**
Plasmid-encoded *ybt* **2**
Distribution of KpZM module amongst ICE*Kp* structures and *ybt* lineages **3**
Genetic diversity of the colibactin synthesis locus **3**
Transmission of ICE*Kp* in the *K. pneumoniae* host population **4**
References **4**
Supplementary Tables **5**
Supplementary Figures **18**

ybt* locus diversity in *K. pneumoniae

The 11 genes comprising the *ybt* locus showed much diversity across the number of unique alleles (n=41-195), nucleotide divergence (0.2%-1.6%) and Simpson's diversity (0.80-0.99) (**Table S3**). The *irp1* and *irp2* genes had the greatest number of alleles; this could be explained by their lengths, as their nucleotide divergence was within the range of other genes (**Table S3**) and nucleotide variation was distributed along the length of the genes (**Fig. S1**).

Phylogenetic relationships between the translated amino acid sequences (for the 295 YbSTs that do not carry frameshift mutations) revealed general concordance between the lineages observed at both the nucleotide and amino acid level (**Fig. S4**). Notably there was little to no differentiation in the predicted translated sequences for *ybt 5* and 6; *ybt* 8 and 9*;* or amongst *ybt 13 – 16* (**Fig. S4**), however we maintain the distinction of these nucleotide-defined lineages as they were each associated with different ICE*Kp* structures. The position of *ybt* 17 is different in the two trees due to a recombination affecting *irp1* (**Fig. S2**), since recombinant sites were filtered from the DNA alignment but not the amino acid alignment.

Plasmid-encoded *ybt*

No chromosomal insertion site could be identified in genomes carrying *ybt* 4 (**Fig. 1**). Inspection of the *de novo* assemblies of these genomes revealed that in all cases, contigs containing the *ybt* locus also harboured common *K. pneumoniae* plasmid sequences including the FIB_K *repA* (plasmid replication) and *sopAB* (plasmid partitioning) genes. It was not possible to resolve complete circular plasmid sequences from the short-read assemblies, however inspection of the assembly graphs showed that the *ybt* 4-encoding contigs were disconnected from the chromosomal contigs, consistent with a plasmid location. To confirm this, we subjected one of the *ybt* 4 isolates from our own collection (ST2370 strain INF167) to long-read sequencing using a MinION (Oxford Nanopore) device and resolved the complete sequence for a 165 kbp circular plasmid carrying *ybt* 4, FIB_K *repA* and *sopAB,* and the FII_K *rep* and its associated *tra-trb* conjugative transfer region. Annotation of this plasmid and the *ybt*+ contigs from the remaining isolates did not reveal any genes with known AMR-related functions.

The *ybt*+ plasmids were found in a variety of *K. pneumoniae* hosts with distinct chromosomal backgrounds, indicative of plasmid transfer between *K. pneumoniae* sublineages. Interestingly 20 (83%) came from isolates we collected from patients at a single hospital (Melbourne, Australia; 2013-2014) and belonging to 15 different chromosomal lineages, suggestive of transmission of the plasmid between locally co-circulating strains. The other *ybt*+ plasmids were found in isolates from Singapore (n=1, 2014), Australia (n=1, 2001) and the US (n=2, 2013 and 2014).

These results indicate that *ybt* 4 is typically plasmid-encoded in *K. pneumoniae*, providing an alternative mechanism for transfer between *K. pneumoniae* hosts. The *ybt* 4 sequences were distinct from those of other *ybt* lineages found in *K. pneumoniae* (>0.28% nucleotide divergence; maximum 1 shared allele) (**Fig. 1**) and shared closer sequence identity with *ybt* genes found in *Yersinia* species (0.01% nucleotide divergence). The ICE*Kp* integrase and mobilisation genes were absent from the *ybt*+ plasmids, and additional complete plasmid sequences will be required to resolve the mechanisms by which *ybt* 4 was acquired into the plasmid backbone.

Distribution of KpZM module amongst ICE*Kp* structures and *ybt* lineages
As noted in the main text, a ~34 kbp Zn^{2+} and Mn^{2+} metabolism module (KpZM) was identified upstream of six different ICE*Kp* structures (**Fig. 2**). The ybt lineages associated with this module formed three clades within the *ybt* sequence tree (*ybt* 1, *ybt* 11 – 12, and *ybt* 15 – 17; **Fig. 1**), suggesting that KpZM was acquired in the ancestors of each of these three clades and has been maintained during subsequent diversification, which in the latter two clades includes divergence into distinct ICE*Kp* structures by swapping out cargo genes. The KpZM module was also found occasionally in *ybt* 10 and *ybt* 14 loci, consistent with more recent and sporadic acquisitions within these ICE*Kp* structures.

Genetic diversity of the colibactin synthesis locus
The *K. pneumoniae clb* locus (**Fig. 3A**) displayed less genetic diversity compared to that of *ybt* (**Table S7**). The 65 unique CbSTs (**Table S8**) identified from a MLST style analysis of the *clb* genes clustered into three distinct *clb* lineages that each corresponded with a particular *ybt* lineage (**Fig. 3B**). The only exceptions were three isolates with *clb* 2B that had rare YbSTs not assigned to any lineage: ST258 strain UCI91 and ST48 strains WGLW1 and WGLW3 (labelled in **Fig. 1**). Two *ybt*- *clb*+ isolates were observed (both ST23). The corresponding *clb* loci clustered with those from the other ST23 ICE*Kp10 ybt*+*clb*+ isolates and shared the same ICE*Kp10* integration site, suggesting a shared ancestral integration event in ST23 followed by subsequent loss of *ybt*.

The *clbJ* and *clbK* genes were excluded from the MLST and phylogenetic analysis due to a common 4173 bp deletion, which results in an open reading frame fusing the 5' end of *clbJ* with the 3' end of *clbK* (**Fig. 3A, Fig. S5**). Intragenic insertions of IS were also detected in various *clb* genes in some strains (**Table S7**). The *clbJ*/*clbK* deletion was detected sporadically in all *clb* lineages, suggesting it has arisen on multiple independent occasions (**Fig. 3B**). *ClbJ* and *clbK* encode multi-domain proteins of 2166 and 2154 amino acids, respectively, whose functions are not yet characterised (**Fig. S5**). The deletion appears to be mediated by recombination between two copies of a 1480 bp stretch of homologous sequence that occurs with ~95% identity within the *clbJ* and *clbK* genes, which encodes an amino acid adenylation domain (A-domain) that is frequently a component of multi-domain non-ribosomal peptide synthetases. The fusion product ClbJK created by the *clbJ*/*clbK* deletion is a 2440 amino acid protein (**Fig. S5C**) that could potentially be functional, however its effect on colibactin synthesis is not yet known. Frameshift mutations and nonsense mutations were also observed but only in a few strains (n=7)(**Table S7**) compared to the number of mutations observed in *ybt*.

Transmission of ICE*Kp* in the *K. pneumoniae* host population
Most unique ICE*Kp* acquisition events (65%) were identified in a single genome sequence. The frequency of *ybt* carriage and unique *ybt* acquisitions per ST was correlated with the number of genomes observed per ST (R^2=0.71, $p<1x10^{-8}$ for log-linear relationship; see **Fig. 4c**), suggesting that the discovery of novel integrations within lineages is simply a function of sampling. This implies that ICE*Kp* is frequently gained and lost from all lineages, and deeper sampling would continue to uncover further acquisitions and losses. Notably, of the 35 clonal groups that were represented by ≥10 genomes, 30 (86%) included at least one ICE*Kp* acquisition (**Fig.**

4, S7). The five other common clonal groups each consisted mostly of isolates from a localised hospital cluster (ST323, Melbourne; ST490, Oxford; ST512, Italy; ST681, Melbourne; ST874, Cambridge); and we predict that more diverse sampling of these clonal groups would detect ICE*Kp* acquisition events.

References

1. Carniel E, Mazigh D, Mollaret H. Expression of iron-regulated proteins in *Yersinia* species and their relation to virulence. Infect Immun. 1987;55:277–80.
2. De Almeida A, Guiyoule A, Guilvout I, Iteman I, Baranton G, Carniel E. Chromosomal *irp2* gene in *Yersinia*: distribution, expression, deletion and impact on virulence. Microb Pathog. 1993;14:9–21.
3. Pelludat C, Rakin A, Jacobi CA, Schubert S, Heesemann J, Pettenkofer-institut M Von. The Yersiniabactin Biosynthetic Gene Cluster of *Yersinia enterocolitica*: Organization and Siderophore-Dependent Regulation. J Bac. 1998;180(3):538–46.

Supplementary Tables

Table S1. Description and sources of genome data used in this study.
*Genome collections consisting of human isolates not associated with outbreaks but with clinical data available, included in **Table S2**.

DATASET	N	NOTES	REF
**Data from individual *Kp* genomics studies:**			
Bialek *et al.*	36	K1/K2 study (multiple sites)	(1)
Bowers *et al.*	157	CG258 study (US)	(2)
Chung *et al.*	76	Outbreak in Patan Hospital (Kathmandu, Nepal)	(3)
Davis *et al.*	63	Isolates from retail meats and human UTIs (US)	(4)
Deleo *et al.*	69	CG258 study (US)	(5)
Ellington *et al.*	193	Addrenbrookes Hospital (Cambridge, UK)	(6)
*Holt *et al.*	273	Global diversity study (multiple sites)	(7)
*Lee *et al.*	26	PLA study (Singapore)	(8)
Onori *et al.*	16	ST258 study at the Circolo Hospital and Macchai Foundation hospital (Varese, Italy)	(9)
Stoesser *et al.* (2013)	69	Isolates from John Radcliffe Hospital (Oxford, UK)	(10)
Stoesser *et al.* (2014)	54	Outbreak in Patan Hospital (Kathmandu, Nepal)	(11)
Struve *et al.*	67	ST23 study (multiple sites)	(12)
Wand *et al.*	33	Pre-antibiotic era strains isolated1917-1949 (UK, Murray Collection)	(13)
*Wyres *et al.*	486	Diverse hospital isolates (Australia), including the genomes from a gastrointestinal carriage study by Gorrie *et al.*	(14, 15)

Data from genome databases:			
NCTC3000	83	phe-culturecollections.org.uk/collections/nctc.aspx	
PATRIC	797	patricbrc.org	

References:

1. Bialek-davenet S, Criscuolo A, Ailloud F, Passet V, Jones L, Garin B, et al. Genomic Definition of Hypervirulent and Multidrug-Resistant *Klebsiella pneumoniae* Clonal Groups. Emerg Infect Dis. 2014;20(11):1812–20.
2. Bowers JR, Kitchel B, Driebe EM, MacCannell DR, Roe C, Lemmer D, et al. Genomic analysis of the emergence and rapid global dissemination of the clonal group 258 *Klebsiella pneumoniae* pandemic. PLoS One. 2015;10(7):1–24.
3. Chung The H, Karkey A, Pham Thanh D, Boinett CJ, Cain AK, Ellington M, et al. A high-resolution genomic analysis of multidrug-resistant hospital outbreaks of *Klebsiella pneumoniae*. EMBO Mol Med. 2015;7(3):227–239.
4. Davis GS, Waits K, Nordstrom L, Weaver B, Aziz M, Gauld L, et al. Intermingled *Klebsiella pneumoniae* Populations between Retail Meats and Human Urinary Tract Infections. Clin Infect Dis. 2015; 61:892–899.
5. Deleo FR, Chen L, Porcella SF, Martens C a, Kobayashi SD, Porter AR, et al. Molecular dissection of the evolution of carbapenem-resistant multilocus sequence type 258 *Klebsiella pneumoniae*. Proc Natl Acad Sci USA. 2014;111(13):4988–4993.
6. Follador R, Heinz E, Wyres KL, Ellington MJ, Kowarik M, Holt KE, et al. The diversity of *Klebsiella pneumoniae* surface polysaccharides. Microb Genomics. 2016;2.
7. Holt KE, Wertheim H, Zadoks RN, Baker S, Whitehouse CA, Dance D, et al. Genomic analysis of diversity, population structure, virulence, and antimicrobial resistance in *Klebsiella pneumoniae*, an urgent threat to public health. Proc Natl Acad Sci USA. 2015;112(27):E3574-81.
8. Lee IR, Molton JS, Wyres KL, Gorrie C, Wong J, Hoh CH, et al. Differential host susceptibility and bacterial virulence factors driving *Klebsiella* liver abscess in an ethnically diverse population. Sci Rep. 2016;6:29316.
9. Onori R, Gaiarsa S, Comandatore F, Pongolini S, Brisse S, Colombo A, et al. Tracking nosocomial *Klebsiella pneumoniae* infections and outbreaks by whole-genome analysis: Small-scale Italian scenario within a single hospital. J Clin Microbiol. 2015;53(9):2861–8.
10. Stoesser N, Batty EM, Eyre DW, Morgan M, Wyllie DH, Del Ojo Elias C, et al. Predicting antimicrobial susceptibilities for *Escherichia coli* and *Klebsiella pneumoniae* isolates using whole genomic sequence data. J Antimicrob Chemother. 2013;68(10):2234–44.
11. Stoesser N, Giess A, Batty EM, Sheppard AE, Walker AS, Wilson DJ, et al. Genome sequencing of an extended series of NDM-producing *Klebsiella pneumoniae* isolates from neonatal infections in a Nepali hospital characterizes the extent of community- Versus hospital- associated transmission in an endemic setting. Antimicrob Agents Chemother. 2014;58(12):7347–57.

12. Struve C, Roe CC, Stegger M, Stahlhut SG, Hansen DS, Engelthaler DM, et al. Mapping the evolution of hypervirulent *Klebsiella pneumoniae*. MBio. 2015;6(4):1–12.
13. Wand ME, Baker KS, Benthall G, McGregor H, McCowen JWI, Deheer-Graham A, et al. Characterization of pre-antibiotic era *Klebsiella pneumoniae* isolates with respect to antibiotic/disinfectant susceptibility and virulence in *Galleria mellonella*. Antimicrob Agents Chemother. 2015;59(7):3966–72.
14. Wyres KL, Wick RR, Gorrie C, Jenney A, Follador R, Thomson NR, et al. Identification of *Klebsiella* capsule synthesis loci from whole genome data. Microb Genomics. 2016;
15. Gorrie CL, Mirceta M, Wick RR, Edwards DJ, Strugnell RA, Pratt N, et al. Gastrointestinal carriage is a major reservoir of *K. pneumoniae* infection in intensive care patients. Clin Infect Dis. 2017;cix270. do.

Table S2. Frequency of yersiniabactin locus (*ybt*) in *K. pneumoniae* isolated from humans in three previous studies. Carriage, isolates recorded as associated with asymptomatic carriage as opposed to infection; N, total number of isolates; *ybt*, number carrying *ybt*. Statistics reported are odds ratio (OR), 95% confidence interval (CI) and p-value for association between *ybt* and infections of different types vs asymptomatic carriage, calculated using Fisher's exact test.

			Infection vs carriage		
	N	*ybt* (%)	OR	95% CI	p-value
**Carriage**	141	18 (13%)	-	-	-
**Infection**					
Liver abscess	32	26 (81%)	28.6	9.9 - 97.4	8×10^{-14}
Blood	112	42 (38%)	4.1	2.1 - 8.1	6×10^{-6}
Respiratory	70	22 (31%)	3.1	1.5 - 6.8	2×10^{-3}
Urine	236	65 (28%)	2.6	1.4 - 4.9	8×10^{-4}
Wound	21	5 (24%)	1.6	0.4 - 5.2	0.4

Table S3. Summary of function, genetic diversity and mutations observed within *ybt* locus genes.

Gene	Length (bp)	Function	No. alleles	Simpson's Index of diversity	Mean (range) nucleotide divergence (SNPs)	Mean (range) nucleotide divergence (%)	dN/dS	Frameshift mutations	Nonsense mutations
ybtS	1305	Salicylate synthetase<br>• involved in salicylate (S) biosynthesis<br>• Salicylate incorporated into Ybt	47	0.87	20.300(1-41)<br>*20.314(1-41)	1.56(0.08-3.07)<br>*1.56(0.08-3.07)	*0.30	-	-
ybtX	1281	Major facilitator superfamily protein<br>• Inner membrane protein<br>• Exact role(s) unknown	60	0.90	20.305(1-54)<br>*19.569(1-41)	1.59(0.08-4.22)<br>*1.53(0.08-3.20)	*0.28	-	-
ybtQ	1803	ATPase/ABC transporter protein<br>• involved in Fe-Ybt transport	65	0.93	9.5781(1-55)<br>*8.420(1-42)	0.53(0.06-3.05)<br>*0.47(0.06-2.33)	*0.27	1	1
ybtP	1713	ATPase/ABC transporter protein<br>• involved in Fe-Ybt transport	75	0.91	9.7474(1-38)<br>*8.599(1-36)	0.57(0.06-2.22)<br>*0.50(0.06-2.10)	*0.27	1	-
ybtA	960	AraC family transcriptional regulator<br>• Activates and regulates expression of *ybt* locus	44	0.80	5.412(1-18)<br>*5.186(1-18)	0.56(0.10-1.88)<br>*0.54(0.10-1.88)	*0.37	-	-
irp2	6108	Encodes HMWP2<br>• NRPS domains involved in non-ribosomal peptide synthesis<br>• Forms part of HMWP2-HMWP1 assembly complex<br>• Accepts adenylated salicylate<br>• Forms two thiazoline rings	193	0.99	20.654(1-146)<br>*19.685(1-93)	0.34(0.02-2.39)<br>*0.32(0.02-1.52)	*0.33	20	18
irp1	9492	Encodes HMWP1<br>• Contains polyketide/fatty acid synthase and modified NRPS domains<br>• Involved in release of complete Ybt product from assembly complex	195	0.99	37.821(1-211)<br>*35.799(1-142)	0.40(0.01-2.22)<br>*0.38(0.01-1.50)	*0.42	12	4
ybtU	1101	Thiazolinyl-S-HMWP1 reductase<br>• Reduces thiazoline ring to form thiazolidine<br>• Cyclizes and condenses second thizaoline ring onto	49	0.81	7.011(1-28)<br>*6.318(1-27)	0.64(0.09-2.54)<br>*0.57(0.09-2.45)	*0.32	1	1

		HMWP1 NRPS domain							
ybtT	804	Thioesterase enzyme<br>• Proofreading enzyme<br>• Removal of abnormal molecules	41	0.90	5.821(1-21)<br>*5.282(1-16)	0.72(0.12-2.61)<br>*0.66(0.12-1.99)	*0.53	1	-
ybtE	1578	Thioesterase enzyme<br>• Adenylates salicylate (A-S)<br>• transfers A-S onto HMWP2	62	0.87	6.797(1-36)<br>*5.927(1-22)	0.43(0.06-2.28)<br>*0.38(0.06-1.39)	*0.51	1	1
fyuA	2022	Outer membrane receptor<br>• Receptor for uptake of Fe-Ybt	102	0.87	5.921(1-106)<br>*4.082(1-18)	0.29(0.05-5.24)<br>*0.20(0.05-0.89)	*0.56	3	2

*asterisks indicate values excluding ybt alleles from the most divergent sequence of *ybt* (YbST314; ybtS_68, ybtX_74, ybtQ_79, ybtP85, ybtA_53, irp2_214, irp1_215, ybtU_58, ybtT_50, ybtE_100 and fyuA_105).

Table S4. Isolates used in this study

(See tab titled 'Table S4' in Excel spreadsheet titled 'Supplementary Data')

Table S5. Yersiniabactin sequence types (YbSTs) and corresponding alleles.

(See tab titled ‘Table S5’ in Excel spreadsheet titled ‘Supplementary Data’)

Table S6. Description of ICE*Kp* variants.

ICE*Kp*	YbST lineages	Length (kbp)	Unique genes in variable regions*	Comments	Accession number#
ICE*Kp1*	2	76	**Middle region:**<br>Virulence associated proteins VagC, VagD, Salmochelin genes *iroN, iroB, iroC, iroD*, Drug/metabolite transporter permease. LuxR family transcriptional regulator. Regulator of mucoid phenotype *rmpA*. SAM-dependent methyltransferase.<br>3 Transposases.<br>2 Hypothetical proteins.<br>**3' region:**<br>Thiamine biosynthesis protein ThiF,<br>DNA binding protein,<br>4 Hypothetical proteins	- First described in *Kp* strain NTUH-K2044 (Lin *et al.* 2008)<br>- Referred to as a 'Group IV' genomic island (Marcoleta *et al.* 2016)	KY454627
ICE*Kp2*	10, 13, 14	62	Thymidylate synthase,<br>Adenylate kinase,<br>TIR domain protein,<br>9 Hypothetical proteins		Ellington *et al.* (49), accession number ERR314530
ICE*Kp3*	8, 9	65	Restriction endonuclease,<br>DUF4917 domain containing protein,<br>ATP/GTP phosphatase,<br>Reverse transcriptase,<br>DDE endonuclease,<br>5 Hypothetical proteins	- BLAST: 99% identity to *E. coli* Co6114<br>- Referred to as a 'Group VI' genomic island (Marcoleta *et al.* 2016)	KY454628
ICE*Kp4*	10	58	Transposase,<br>ABC transporter,<br>Type I restriction endonuclease,<br>DNA methyltransferase,<br>Hypothetical protein	- BLAST: 99% identity to *E. coli* ED1a	KY454629
ICE*Kp5*	6, 14	66	DEAD/DEAH box helicase,<br>Thiamine biosynthesis protein ThiF,<br>2 Patatin-like phospholipases,	- BLAST: 99% identity to *Enterobacter hormachei* 05-545	KY454630

			6 Hypothetical proteins	- Referred to as a 'Group V' genomic island (Marcoleta *et al.* 2016)	
ICE*Kp6*	5	69	Helicase,<br>Kinetochore protein,<br>DNA cytosine methylase,<br>Low calcium response locus protein S,<br>Transposase,<br>4 Hypothetical proteins		KY454631
ICE*Kp7*	7	87	mRNA endoribonuclease LS,<br>Chromosome partition protein ParA,<br>ATPase,<br>LPS kinase,<br>Tellurite resistance protein TerY,<br>12 Hypothetical proteins		KY454632
ICE*Kp8*	3	58	Nucleotidyltransferase,<br>Helicase,<br>2 Hypothetical proteins		strain BIDMC21 (1328416.3), (PATRIC)
ICE*Kp9*	8	57	DNA methyltransferase,<br>3 Hypothetical proteins		KY454633
ICE*Kp10*	1, 12 and 17	138	Colibactin synthesis locus (*clbQ, clbP, clbO, clbN, clbM, clbL, clbK, clbJ,*<br>*clbI, clbH, clbG, clbF, clbE, clbD, clbC, clbB, clbA*).<br>Transposase IS3/IS911 family,<br>Integrase catalytic subunit	- BLAST:<br>99% identity to *Citrobacter koseri* ATCC BAA-898 and a number of *Enterobacter aerogenes* strains<br><br>- Referred to as KPHPI208 in *Kp* strain 1084 (Lai *et al.* 2014)<br>- Referred to as GI-I in *Kp* strain Kp52.145 (Lery *et al.* 2014)<br>- Referred to as a 'Group III' genomic island (Marcoleta *et al.*	KY454634

				2016)	
ICE*Kp11*	15	92	DNA protecting protein dprA,<br>8 Hypothetical proteins		KY454635
ICE*Kp12*	9, 10, 14, 16	97	Exonuclease SbcC,<br>DNA helicase UvrD,<br>ATP-dependent endonuclease,<br>5 Hypothetical proteins		KY454636
ICE*Kp13*	Not in main lineages	65	DEAD/DEAH box helicase,<br>Chromosome partition protein Smc,<br>Serine protease,<br>Metallobetalactamase superfamily protein,<br>5 Hypothetical proteins		KY454637
ICE*Kp14*	Not in main lineages		**Middle region:**<br>Taurine catabolism dioxygenase TfdA,<br>Histidine ammonia lyase,<br>Histidinol-phosphate aminotransferase,<br>Threonyl and alanyl tRNA synthetase domain protein,<br>Lactate dehydrogenase,<br>ATP-grasp domain-containing protein,<br>MFS transporter,<br>DNA-binding protein,<br>Phage conjugal plasmid C4 type zinc finger protein,<br>3 Hypothetical proteins<br>**3' region:**<br>LysR family transcriptional regulator,<br>Alcohol dehydrogenase,<br>ATP-binding protein,<br>DNA helicase UvrD,<br>ATPase,<br>3 Hypothetical proteins		KY454638

*unique genes in the 3' variable region unless otherwise specified
#Accession numbers for the ICE*Kp* references assembled from in-house sequencing data have been provided, with the exception of ICE*Kp2* and ICE*Kp8,* which were identified or assembled from publicly available sequence data as listed.

Table S7. Summary of function, genetic diversity and mutations observed within *clb* locus genes.

Gene	Length (bp)	Function/product	No. alleles	Mean (range) nucleotide divergence (SNPs)	Mean (range) nucleotide divergence (%)	Simpson's Index of diversity	dN/dS	Frameshift mutations	Nonsense mutations	Transposase insertion
clbA	735	Modification/accessory enzyme: Phosphopantetheinyl transferase – primes NRPS-PKS enzymes	6	3(1-5)	0.41(0.14-0.68)	0.53	0.43	1	-	2
clbB	9621	Clb Biosynthesis: NRPS/PKS hybrid	33	13.7235(1-19)	0.14(0.01-0.20)	0.81	0.37	1, 2*	-	177
clbC	2601	Clb Biosynthesis: PKS	7	4.8571(1-8)	0.19(0.04-0.31)	0.16	0.13	-	-	4
clbD	867	Modification/accessory enzyme: hydroxylacyl-CoA dehydrogenase	6	3.4667(1-6)	0.40(0.12-0.69)	0.16	0.19	-	1	-
clbE	249	Clb Biosynthesis	6	1.6(1-2)	0.64(0.40-0.80)	0.17	0.17	1	-	-
clbF	1131	Modification/accessory enzyme: dehydrogenase	6	2.4(1-4)	0.21(0.09-0.35)	0.50	0.15	-	-	2
clbG	1269	Clb Biosynthesis	5	2.2(1-4)	0.17(0.08-0.32)	0.05	0.67	-	-	1
clbH	4797	Clb Biosynthesis: NRPS	13	7.6667(1-15)	0.16(0.02-0.31)	0.49	0.33	1	-	7
clbI	3033	Clb Biosynthesis: PKS	11	5.4182(1-10)	0.18(0.03-0.33)	0.17	0.68	1	-	2
clbJ	6501	Clb Biosynthesis: NRPS	NA	NA	NA	NA	NA	NA	NA	NA
clbK	6465	Clb Biosynthesis: NRPS/PKS hybrid	NA	NA	NA	NA	NA	NA	NA	NA
clbL	1464	Modification/accessory enzyme: Amidase	6	3.6667(1-8)	0.25(0.07-0.55)	0.17	0.4	-	-	2
clbM	1440	Putative transporter	6	4(1-7)	0.28(0.07-0.49)	0.15	0.31	1	-	-
clbN	4368	Clb Biosynthesis: NRPS	11	10.8(1-20)	0.25(0.02-0.46)	0.17	0.23	-	-	-
clbO	2460	Clb Biosynthesis: PKS	10	4.7556(1-9)	0.19(0.04-0.37)	0.19	0.42	-	-	1
clbP	1506	Modification/accessory enzyme: Membrane bound D-asparagine peptidase involved in maturation of non-ribosomal peptides/activation of colibactin	8	2.7857(1-5)	0.18(0.07-0.33)	0.17	1.22	-	-	1
clbQ	723	Clb Biosynthesis	3	2.6667(1-4)	0.37(0.14-0.55)	0.14	0.45	-	-	1

*NOTE. 2 *clbB* alleles with 3- and 6 bp insertions respectively that does not cause a frameshift mutation

Table S8. Colibactin sequence types (CbSTs) and corresponding alleles

(See tab titled 'Table S8' in Excel spreadsheet titled 'Supplementary Data')

Table S9. Description of *ybt* loci detected in *Enterobacteriaceae* bacteria

(See tab titled 'Table S9' in Excel spreadsheet titled 'Supplementary Data')

Supplementary Figures

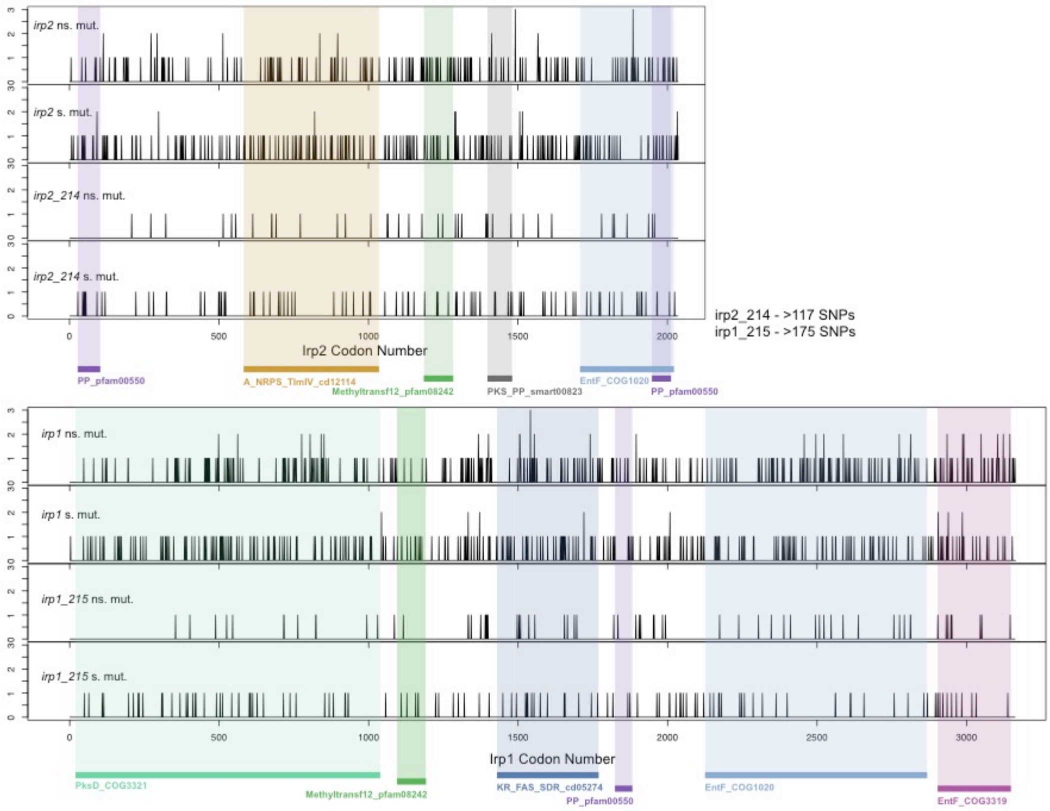

Figure S1. Number and distribution of non-synonymous (ns. mut.) and synonymous mutations (s. mut.) across the translated Irp2 and Irp1 peptides. The number of mutations are shown on the y-axis of each panel. Non-synonymous and synonymous mutations observed across the *irp2* and *irp1* alleles found in the most divergent sequence of *ybt* are shown on separate plots and labelled accordingly. Key domains within each peptide are highlighted and labelled with domain names and accession numbers.

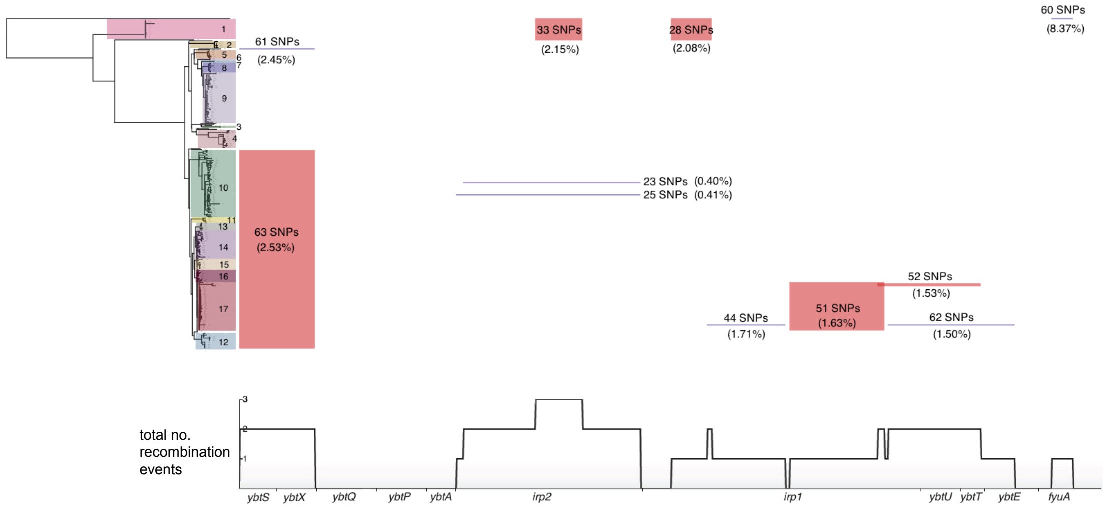

Figure S2. Predicted recombination events in the *ybt* locus.
Recombination events were predicted using Gubbins and are shown as coloured blocks (visualised using Phandango). The number of SNPs introduced (and corresponding % nucleotide divergence) in each recombinant block is indicated. Coordinates along the *ybt* locus and gene boundaries are indicated on the x-axis, with a separate plot showing the total number of recombination events detected. Each row in the plotting area represents a YbST. Phylogenetic relationships between the YbSTs are shown in the tree to the left, which is a midpoint-rooted, recombination-free YbST phylogeny reproduced from **Figure 1**. Colours and numbers on the tree indicate *ybt* lineages as detailed in the text and **Figure 1**.

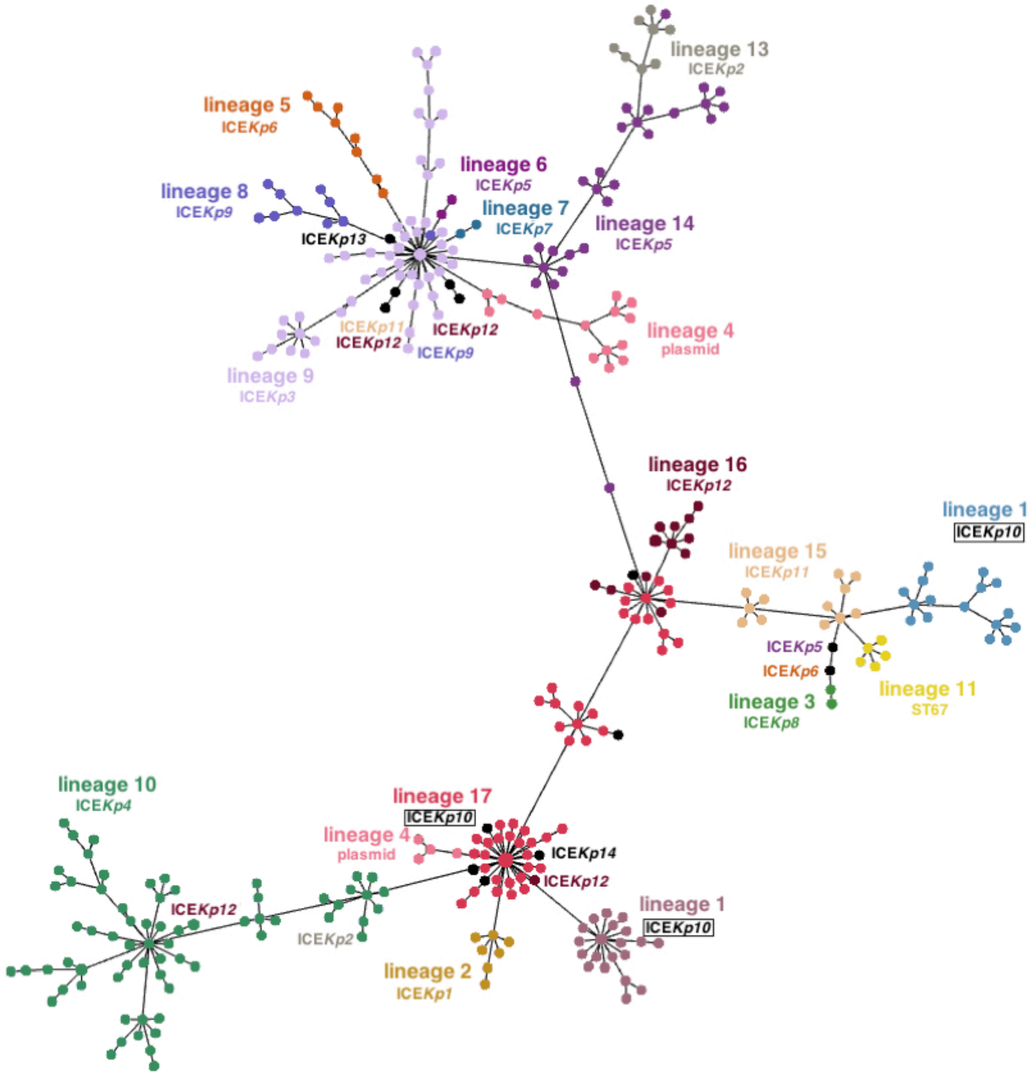

Figure S3. Minimum spanning tree of YbSTs, visualised using PhyloViz. Each node represents a YbST, connections between the nodes indicate allele sharing between YbSTs; nodes are coloured by *ybt* lineages (as defined in **Figure 1**; black indicates no lineage assigned) and labelled with ICE*Kp* structures (as defined in **Figure 2**; the *clb*+ ICEKp10 structure, boxed, is associated with three *ybt* lineages).

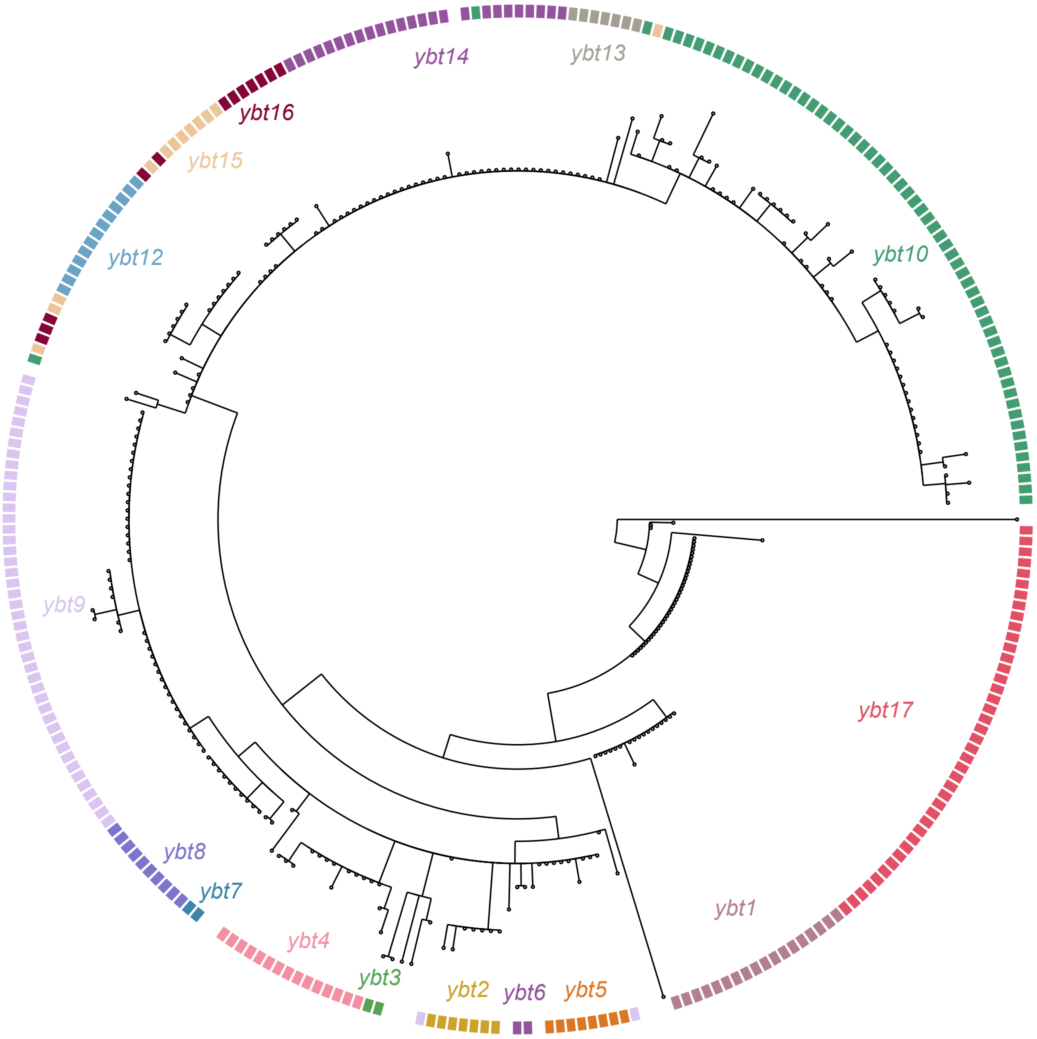

Figure S4. Phylogenetic relationships between the predicted amino acid sequences of 295 YbSTs.
Each leaf represents a translated amino acid sequence for a yersiniabactin sequence type (YbST), excluding those that encode a frameshift mutation (see **Table S4** for strains with these mutations). The corresponding lineages from **Figure 1** are marked on the outer track and labelled accordingly; white = unassigned.

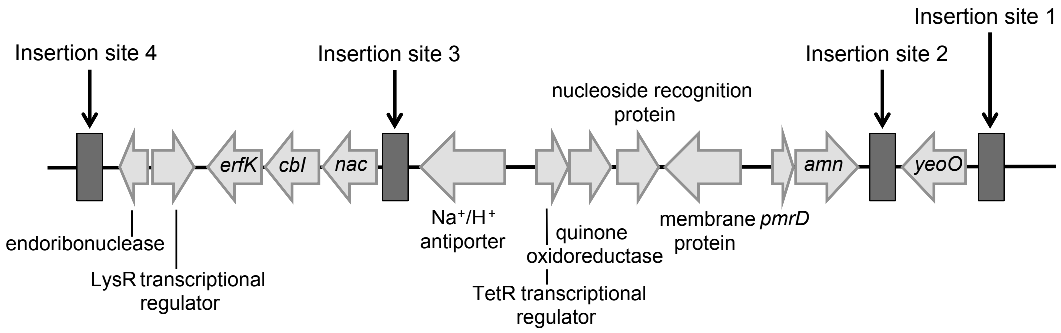

Figure S5. Chromosome region in *K. pneumoniae* containing tRNA-Asn sites that are targeted by yersiniabactin ICE*Kp* elements. The hotspots for insertion of ICE*Kp* and other genomic islands occur within four tRNA-Asn sites, represented by the rectangular blocks, and are marked in the figure. Grey arrows represent coding sequences, labelled by gene symbol or the encoded protein product.

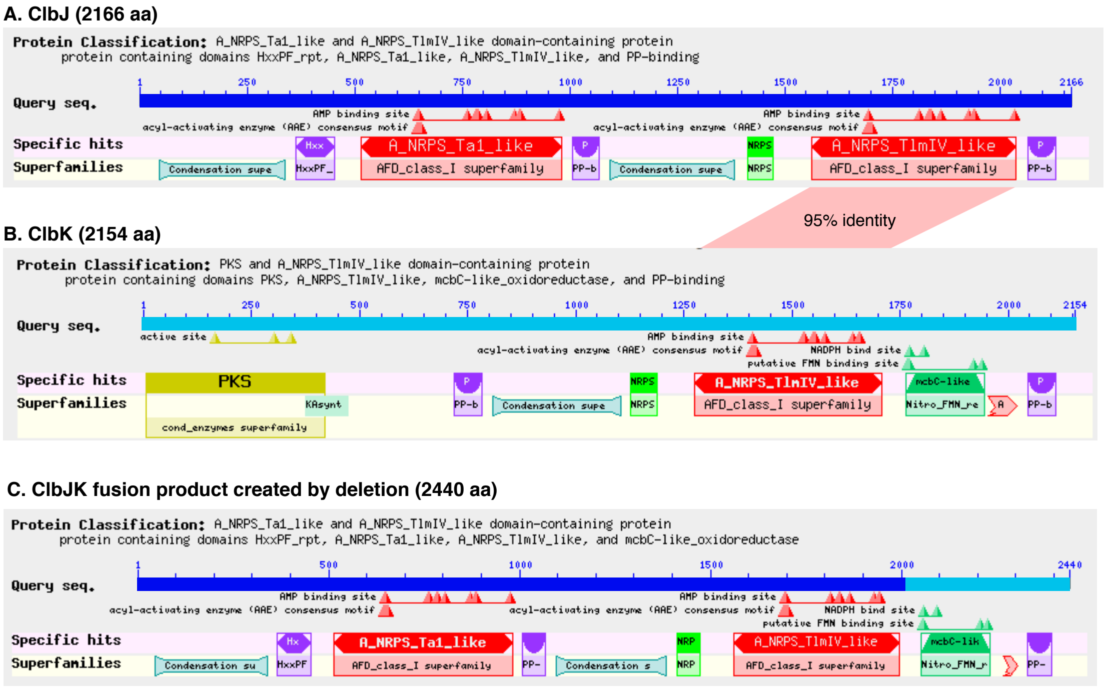

Figure S6. Conserved domains present in the predicted proteins encoded by (A) *clbJ*, (B) *clbK* and (C) the *clbJ/K* deletion. The homologous region shared between the amino acid adenylation domains of *clbJ* and *clbK* is shown. The left hand side and a large portion of the fusion product matches to *clbJ* (dark blue) while the right hand side matches to *clbK* (light blue).

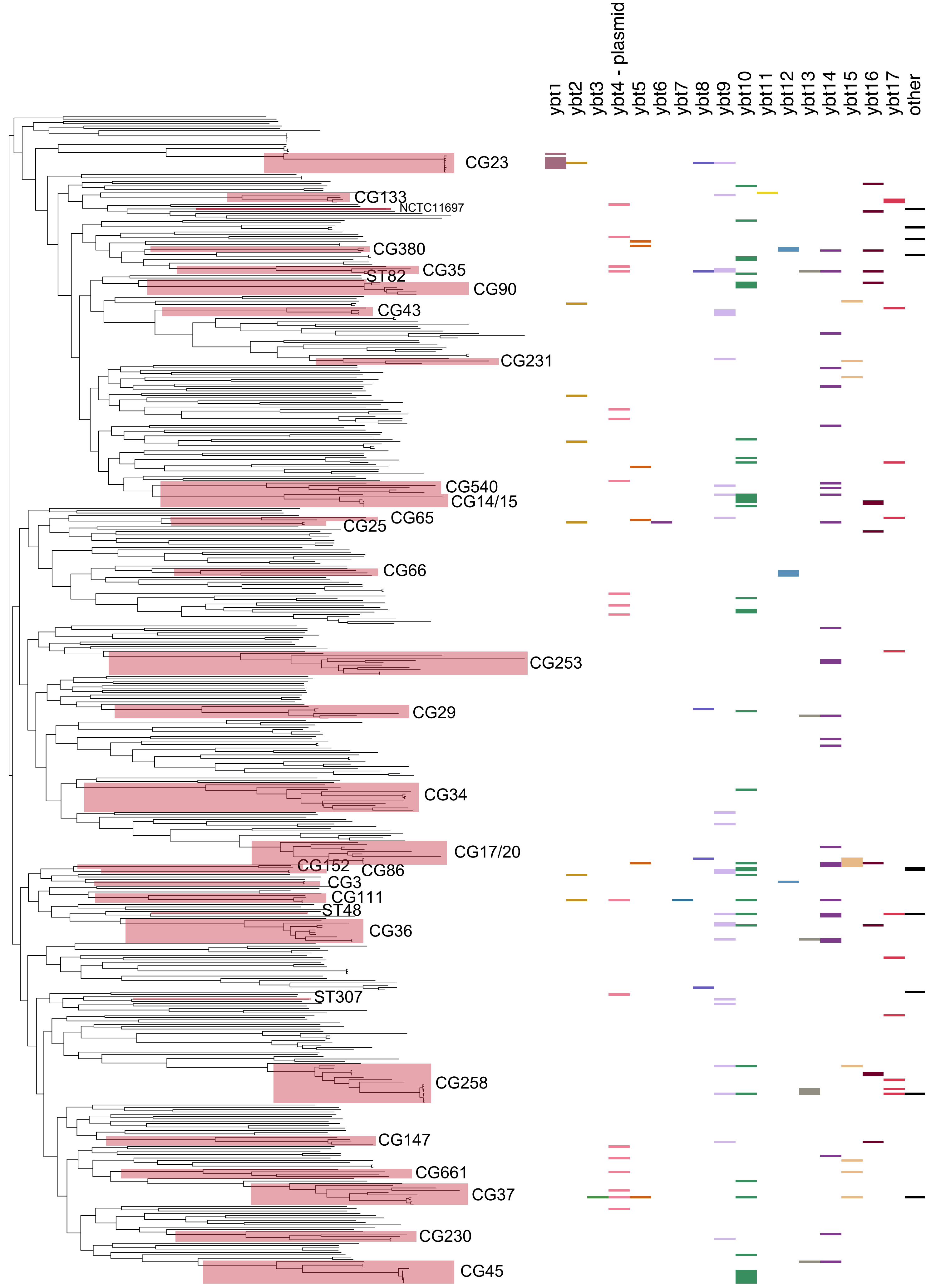
ybt1
ybt2
ybt3
ybt4 - plasmid
ybt5
ybt6
ybt7
ybt8
ybt9
ybt10
ybt11
ybt12
ybt13
ybt14
ybt15
ybt16
ybt17
other
CG23
CG133
NCTC11697
CG380
CG35
ST82
CG90
CG43
CG231
CG540
CG14/15
CG65
CG25
CG66
CG253
CG29
CG34
CG17/20
CG152
CG86
CG3
CG111
ST48
CG36
ST307
CG258
CG147
CG661
CG37
CG230
CG45

Figure S7. Distribution of *ybt* lineages amongst *K. pneumoniae* chromosomal lineages. The tree shown is an outgroup-rooted maximum likelihood phylogeny inferred from recombination-filtered *K. pneumoniae* core gene SNPs. Each tip represents a unique *K. pneumoniae* chromosomal ST; clonal groups with ≥10 genome sequences available for analysis are highlighted and labelled. Strain NCTC11697, which carries the most divergent *ybt* sequence, is also highlighted and labelled. The heatmap indicates which *ybt* lineages were detected within each *K. pneumoniae* ST (the relative abundance of *ybt* lineages within each of the highlighted clonal groups are shown in **Figure 4**).

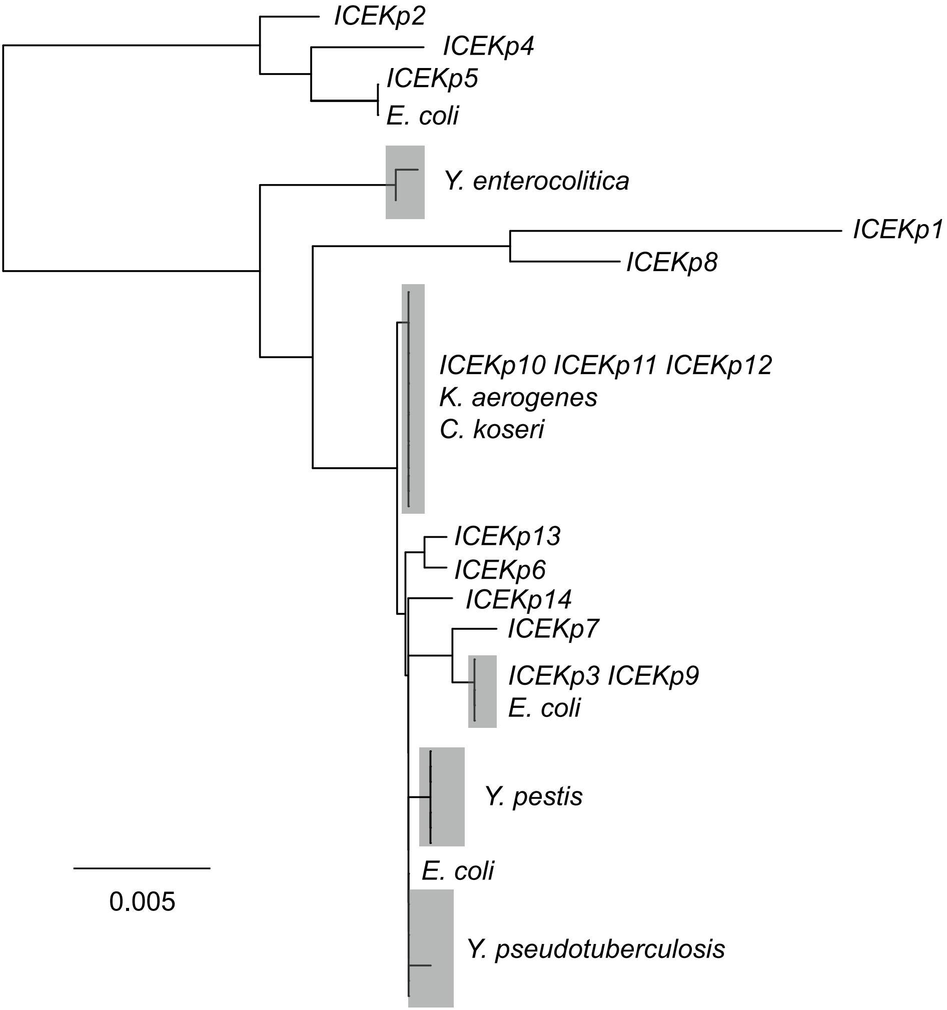
ICEKp2
ICEKp4
ICEKp5
E. coli
Y. enterocolitica
ICEKp1
ICEKp8
ICEKp10 ICEKp11 ICEKp12
K. aerogenes
C. koseri
ICEKp13
ICEKp6
ICEKp14
ICEKp7
ICEKp3 ICEKp9
E. coli
Y. pestis
E. coli
Y. pseudotuberculosis
0.005

Figure S8. Phylogenetic relationships between the *int* found in ICE*Kp* and yersiniae HPI. The tree shown is a neighbour-joining tree of the integrase gene detected in *K. pneumoniae, K. aerogenes, C. koseri* and *E. coli*, and from the HPI in *Y. pseudotuberculosis, Y. pestis* and *Y. enterocolitica*.
